# WDR72 Regulates Microtubule-Associated Vesicular miRNA Export in Ameloblasts During Enamel Maturation

**DOI:** 10.1007/s00223-026-01521-x

**Published:** 2026-04-27

**Authors:** Trang Duong, Jake Ngu, Kaitlin A. Katsura, Pamela DenBesten, Yukiko Nakano

**Affiliations:** 1 Department of Orofacial Science, School of Dentistry and Oral and Craniofacial Science Program, Division of Graduate Education and Postdoctoral Affairs, University of California, 513 Parnassus Ave, HSW860, San Francisco, CA 94143 USA; 2 Department of Orofacial Sciences, School of Dentistry, University of California, San Francisco, CA USA; 3 Center for Oral Health Research, School of Dentistry, University of California, San Francisco, CA USA; 4 Department of Pediatrics, Children’s Hospital of Philadelphia, Philadelphia, PA USA; 5 Institute of Translational Medicine and Therapeutics, University of Pennsylvania, Philadelphia, PA USA

**Keywords:** miRNA, Vesicular miRNA, Microtubule, Regulatory RNA, Ameloblast modulation, Structural genes

## Abstract

Enamel maturation depends on the ability of ameloblasts to undergo repeated cycles of morphological remodeling, a process known as ameloblast modulation. These transitions require precise coordination between cytoskeletal reorganization and vesicular trafficking, yet the intracellular mechanisms that support this coordination remain unclear. Although mutations in the *Wdr72* gene cause enamel defects, the cellular role of WDR72 during enamel maturation has not been defined. Here, we investigated whether WDR72 functions within the microtubule-dependent vesicular trafficking machinery and whether disrupting this function alters vesicular-mediated microRNA (miRNA) export during ameloblast modulation. Using *Wdr72* functional knockout (fKO) mouse and cell models, we examined WDR72 function in relation to microtubules, CD63-positive (CD63^+^) multivesicular bodies (MVBs) that carry vesicles destined for extracellular vesicles (EVs) along microtubules, and miRNA export via EVs. We found that WDR72 associates with microtubules in ameloblast-lineage cells. Loss of WDR72 function disrupts this association without preventing MVB engagement with microtubules. Instead, *Wdr72*-fKO cells exhibit intracellular accumulation of CD63^+^ vesicles, indicating impaired MVB transit or vesicle export, accompanied by reduced vesicle-associated miRNA export. Integration of miRNA and mRNA expression profiles from *Wdr72*-deficient enamel organs revealed an inverse relationship between accumulated circulating miRNAs and reduced levels of their predicted target transcripts, which are enriched for cytoskeletal genes essential for maintaining ameloblast morphology and polarity. Together, these findings identify WDR72 as a microtubule-associated protein that links vesicular miRNA export to enamel maturation and show that disruption of this pathway impairs ameloblast modulation during enamel mineralization.

**Supplementary Information:**

The online version contains supplementary material available at 10.1007/s00223-026-01521-x.

## Introduction

Dental enamel formation depends on the ability of ameloblasts to undergo repeated, highly coordinated morphological transitions during the maturation stage. As ameloblasts alternate between ruffle-ended and smooth-ended states, they must rapidly reorganize their cytoskeleton, remodel membrane domains, and traffic vesicles to precise intracellular and extracellular destinations. These cyclical transitions, collectively referred to as ameloblast modulation, are essential for regulating ion transport, matrix degradation, and crystal growth, and failure of this process results in hypo-mineralized enamel [1–3]. Despite its importance, the intracellular mechanisms that support repeated rounds of cytoskeletal remodeling and vesicle trafficking during ameloblast modulation remain incompletely defined.

Microtubules play a central role in vesicle transport in polarized epithelial cells by serving as directional tracks that guide vesicles toward specific docking and export sites [4–6]. During enamel maturation, microtubule-dependent vesicle transport is thought to be particularly critical, as ameloblasts repeatedly reorganize their apical and basolateral domains while maintaining continuous vesicle flux [7]. However, how microtubule organization and function are regulated in ameloblasts during modulation, and how defects in this system contribute to enamel pathology, remains poorly understood.

One protein strongly implicated in this process is WDR72, a WD-repeat–containing protein enriched in maturation-stage ameloblasts [8]. Loss-of-function mutations in the *Wdr72* gene, referred to in this study as *Wdr72* fKO, cause a condition called amelogenesis imperfecta in humans and mice, characterized by defective enamel mineralization and impaired ameloblast function [7, 9–11]. Prior studies have demonstrated that WDR72 deficiency disrupts vesicle transport during enamel maturation [7], and alters cytoskeletal organization, including direct association with intermediate filament networks such as keratins [12]. These findings indicate that WDR72 is required for intracellular organization during ameloblast modulation, yet its precise molecular role within the cytoskeletal and vesicle trafficking machinery has remained unresolved.

Notably, transcriptomic analyses of *Wdr72* fKO enamel organs have reported reduced expression of *Tppp*, a Tubulin Polymerization Promoting Protein essential for microtubule stability [12], suggesting that WDR72 may influence microtubule organization directly or indirectly. Whether WDR72 physically associates with microtubules, and whether such an association is required for microtubule-dependent vesicle trafficking during ameloblast modulation, has not been determined.

In addition to proteins and ions, vesicles also transport regulatory RNAs, including microRNAs (miRNAs). MiRNAs are small non-coding RNAs that post-transcriptionally regulate gene expression and can be selectively exported via extracellular vesicles. Vesicle-mediated miRNA export, also known as circulating miRNA, plays an important role in maintaining intracellular RNA homeostasis [13], cell–cell communication [14], and modulating cytoskeletal organization, adhesion, and signaling pathways in diverse tissue types [15, 16]. When vesicle export is impaired, circulating miRNAs can become aberrantly retained within the cell, where they may repress gene networks critical for cellular structure and function. Despite growing recognition of circulating miRNA trafficking in other epithelial systems, its role during enamel maturation and ameloblast modulation has not been explored.

Here, we investigated whether WDR72 functions as a microtubule-associated regulator that links vesicle trafficking to miRNA homeostasis during ameloblast modulation. Using our *Wdr72* fKO mouse model and an ameloblast-lineage cell (ALC) system, we combined super-resolution microscopy, quantitative vesicle analysis, miRNA and mRNA sequencing, and integrative bioinformatic approaches. We demonstrated that WDR72 associates directly with microtubules and that loss of this association disrupts vesicle export without preventing vesicle–microtubule engagement. Impaired vesicle trafficking led to intracellular accumulation of vesicles and coordinated retention of vesicular miRNAs, resulting in repression of cytoskeletal, adhesion, and trafficking-related gene networks essential for ameloblast modulation.

Together, these findings established WDR72 as a microtubule-associated regulator of vesicle-mediated miRNA export and identified vesicle-dependent miRNA trafficking as a previously unrecognized mechanism controlling cytoskeletal and transcriptional programs during enamel maturation.

## Materials and Methods

### Animals and Genotyping

*Wdr72* mutant mice, which mimic human hypo-maturation amelogenesis imperfecta, were maintained in the UCSF vivarium under pathogen-free conditions. This strain was previously reported and validated for enamel maturation defects [17]. Genotyping was conducted from tail biopsies using PCR-based assays. Both male and female mice were included. Molars from postnatal day 12 (P12) mice were dissected to collect the enamel organ. All animal procedures adhered to UCSF Institutional Animal Care and Use Committee (IACUC) standards and received approval under our lab protocol. The animals were housed on a 12-h light–dark cycle with free access to food and water.

### *Wdr72* fKO ALC Cell Culture

*Wdr72* fKO ameloblast-lineage cell (ALC) line was previously created in our laboratory using CRISPR-Cas9–mediated guide RNAs targeting exon-12 with mouse ALCs (a kind gift from Dr. Toshihiro Sugiyama, Akita University, Japan and Dr. John Bartlett, Forsyth Institute, Boston, Massachusetts) [7, 18]. Single-cell clones were expanded and validated by Sanger sequencing and Western blot. *Wdr72* fKO and wild-type (WT) ALCs were cultured in DMEM/F12 supplemented with 10% fetal bovine serum (Gibco) and 1% penicillin–streptomycin at 37 °C, 5% CO_2_. Passage 27 was used for all experiments. For qRT-PCR assays, cells were plated at equal densities and harvested at 70–80% confluence to extract total RNA. For vesicle-secretion assays, cells were cultured in exosome-depleted medium (System Biosciences) for 48 h before collecting conditioned media.

### Vesicle Isolation and Vesicular miRNA Extraction

Extracellular vesicles (EVs) were collected from WT and *Wdr72* fKO ALCs cultured for 24 h in DMEM supplemented with 2% TCM Defined Serum Replacement (MP Biomedicals, Cat. No. ICN2010026) and 1% penicillin–streptomycin. For each genotype, four T75 flasks served as biological replicates. Conditioned medium (10 mL per flask) was clarified by low-speed centrifugation, and EVs were isolated using the miRCURY® Exosome Cell/Urine/CSF Kit (Qiagen, Cat. No. 76743) according to the manufacturer’s protocol. Pelleted vesicles were resuspended in the supplied buffer and stored at + 20 °C before RNA extraction. Vesicular RNA was extracted using the Exosomal RNA Isolation Kit (Norgen Biotek, Cat. No. NC1932540). Simultaneously, matched flasks were used to isolate intracellular RNA and DNA for normalization purposes, with RNA purified using TRIzol (Thermo Fisher) and Direct-zol (Zymo Research), and DNA purified by ethanol precipitation. Vesicular miRNAs were reverse-transcribed using the miRCURY LNA RT kit (Qiagen).

### Immunofluorescent Staining

After fixation with 0.5% glutaraldehyde in microtubule-stabilizing buffer (MTSB), followed by quenching with 0.1% sodium borohydride, ALCs were blocked with 2% bovine serum albumin (BSA) in PBS, followed by primary antibody incubation for overnight at 4 °C and secondary antibody incubation for 1 h at room temperature. Primary and secondary antibodies used were: anti-α-tubulin/TUBA1A (Millipore Sigma, clone YL1/2; # MAB1864) with Alexa Fluor 647–conjugated secondary antibody, anti-pan-cytokeratin (Biotium, # BNC680999) with CF568-conjugated secondary antibody, anti-CD63 (BioLegend, # 143,918) with Alexa Fluor 488–conjugated secondary antibody, and anti-WDR72 (Sigma-Aldrich, # 1061–1075) with CF583R-conjugated anti-rabbit IgG secondary antibody.

### Super-Resolution Microscopy (ONI Nanoimager)

Fixed ALCs were imaged on the ONI Nanoimager using dSTORM acquisition. Imaging settings followed the manufacturer-optimized parameters shown in the ONI report: 488 nm laser at 60%, 561 nm at ~ 58%, 640 nm at 60–73%, 10,000 frames, 20 ms frame rate, HILO/TIRF illumination angles 51–54° (https://oni.bio/resources/resource-library/?application=immuno-oncology).

Images were analyzed in CODI (ONI, https://alto.codi.bio/) for cluster segmentation, biomarker co-localization, and localization density using the parameters shown in the ONI report. Localization refers to the number of fluorophores that blink; the number of localizations in each cluster was based on signal strength, cluster masks, and a counting radius of 50 nm. ROIs were defined per cell, and at least three cells per genotype were analyzed.

Super-resolution dSTORM imaging was performed in ameloblast-lineage cells (ALCs) rather than enamel organ tissue due to technical constraints related to tissue thickness and optical complexity. Because tissue dSTORM data did not meet the resolution required for reliable quantitative analysis. ALCs therefore provided a technically appropriate and biologically relevant system to resolve WDR72-microtubule interactions at nanoscale resolution.

### miRNA-seq

Enamel organs were dissected from four postnatal day 12 (P12) first molars per mouse, pooled for each biological replicate. Four WT and four *Wdr72* fKO biological replicates were processed. Total RNA enriched for small RNAs was extracted with the miRNeasy Mini Kit (Qiagen). Library preparation was conducted at the UCSF CAT facility and sequenced as pair-end 50-bp reads.

Quality control was performed using FastQC v0.12.1. Adapter trimming, read collapsing, length filtering, and alignment followed this workflow:

- cutadapt v4.4 for adapter trimming (minimum length 18 nt, mismatch overlap 5)
- fastx_collapser for collapsing identical reads
- filtering for 18–25 nt species
- alignment to mmu mature miRNAs (miRBase v22) using bowtie v1.3.1
- SAM/BAM processing with samtools v1.17
- read counting via samtools idxstats, then merging into an expression matrix

A total of 8 miRNA libraries (4 WT, 4 fKO) were processed.

### Bioinformatic Analyses

miRNA target prediction was performed using miRDB (v6.0, https://mirdb.org/cgi-bin/search.cgi). Differential expression analyses were conducted in R (R v4.3.1) using packages of edgeR, DESeq2, and ggplot2. PCA and normalization scripts followed the R code included in the project workflow. mRNA–miRNA integration analyses were performed by correlating downregulated transcripts (mRNA-seq) with upregulated intracellular miRNAs.

Super-resolution microscopy cluster statistics were exported from CODI (ONI software, 2025 build) and processed in Python (Python v3.10, NumPy, pandas).

### Quantitative RT-PCR

Quantitative RT-PCR (qRT-PCR) of miRNA was performed using the miRCURY LNA SYBR Green PCR Kit (Qiagen) on the QuantStudio 6Pro system (Thermo Fisher). Each sample included three technical replicates and three biological replicates per genotype. U6 snRNA was used as the reference. Relative expression was determined using the ΔΔCt method.

### Statistical Analysis

Data were analyzed using GraphPad Prism (v10.2) or R. Two-tailed unpaired Welch’s t-tests were performed for comparisons between WT and *Wdr72* fKO groups unless otherwise indicated. Data are shown as mean ± SD. Significance was set at *p* < 0.05.

## Results

### WDR72 is Directly Associated with Microtubules

**Fig. 1:**
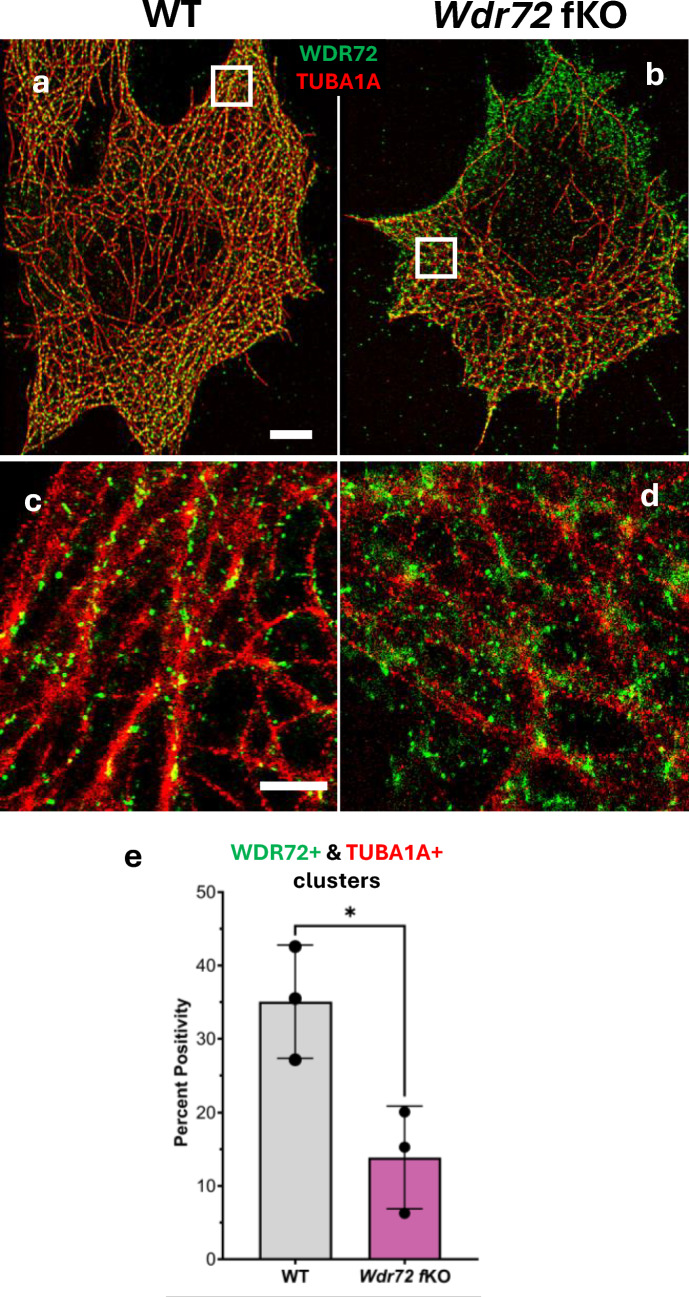
WDR72 associates with microtubules, and its loss reduces microtubule-associated WDR72 clusters: **a**–**d** Representative super-resolution images of ALCs immunostained for WDR72 (green) and α-tubulin (TUBA1A, red) in WT (**a**) and *Wdr72* fKO (**b**) cells. White boxes indicate regions magnified in panels (**c**–**d**). Scale bar, 25 µm. WDR72 positive signals closely align with the microtubule network in WT cells, whereas this organization is markedly reduced and dispersed in *Wdr72* fKO cells. High-magnification views clearly demonstrate WDR72 localization along microtubule tracks in WT cells (**c**) and the loss of organized WDR72–microtubule association in *Wdr72* fKO cells (**d**). Scale bar, 5 µm. **e** Quantification of WDR72^+^/TUBA1A^+^ colocalized clusters expressed as percent positivity per cell area. WT cells show a significantly higher proportion of WDR72–microtubule clusters compared to *Wdr72* fKO cells, indicating disrupted microtubule engagement upon WDR72 loss. Data are shown as mean ± SD with individual data points representing independent cells. *P* < 0.05

Because resolving protein-microtubule interactions requires nanoscale spatial precision, this analysis was performed in ameloblast-lineage cells optimized for super-resolution imaging. To determine whether WDR72 physically associates with the microtubule network in ameloblasts, we examined the spatial relationship between WDR72 and microtubules using immunofluorescence staining in ameloblast-lineage cells (ALCs) with wild-type (WT) and *Wdr72* functional knockout (*Wdr72* fKO) [7]. Anti-WDR72 and anti-TUBA1A labeling were visualized by ONI Nanoimager super-resolution microscopy. In WT cells, WDR72 exhibited a widespread cytoplasmic distribution that closely aligned with the TUBA1A-positive microtubule lattice (Fig. 1a). High-magnification insets revealed WDR72 puncta along with microtubule tracks (Fig. 1c), indicating a direct association between WDR72 and microtubules. In contrast, *Wdr72* fKO cells showed less organized TUBA1A-positive microtubules network along with a markedly altered WDR72 signal, characterized by diffuse cytoplasmic distribution and substantially reduced co-alignment with microtubules (Fig. 1b and d). This loss of structured co-localization suggests that functional loss of WDR72 disrupts its ability to associate with the microtubule network. Consistent with these observations, quantitative cluster analysis demonstrated a significant reduction in the proportion of WDR72–TUBA1A double-positive clusters in *Wdr72* fKO cells compared with WT (Fig. 1e). Together, these data show that WDR72 physically associates with microtubules in ALCs and that this interaction depends on the intact function of WDR72.

In multiple cells, the WDR72 signal was more concentrated at the cell periphery compared to the cytosol of the fKO cell; however, the clear one-sided enrichment shown in Fig. 1b was not observed in all cells. Since ALCs do not exhibit apico-basal polarity similar to maturation-stage ameloblasts, this asymmetric WDR72 fKO distribution was not regarded as a polarity marker, but rather as the uneven focal plane within this cell.

### CD63-Positive Vesicles Accumulate Intracellularly in *Wdr72*-Functional Knockout Cells

**Fig. 2:**
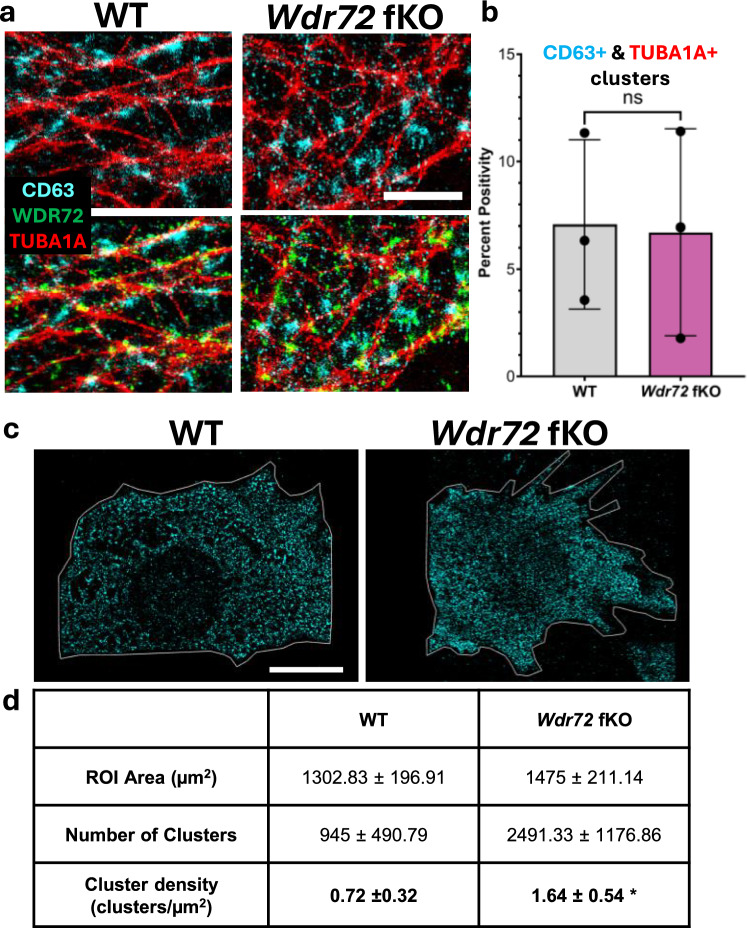
Loss of WDR72 does not alter microtubule association of CD63^+^ vesicles but reduces overall vesicle abundance: **a** 3D view of representative super-resolution images showing CD63^+^ vesicles (cyan), WDR72 (green), and α-tubulin (TUBA1A, red) in ALCs. CD63^+^ vesicles are frequently observed in close proximity to microtubule tracks. Scale bar, 20 µm. **b** Quantification of CD63^+^/TUBA1A^+^ colocalized clusters expressed as percent positivity per cell area in WT and *Wdr72* fKO cells. No significant difference was detected between genotypes (ns), indicating that WDR72 loss does not impair vesicle association with microtubules. Data are shown as mean ± SD with individual data points representing independent cells. **c** Whole-cell super-resolution maps of CD63^+^ vesicle clusters in WT and *Wdr72* fKO ALC. The outlined region of interest (ROI) corresponds to the full projected cell area used for quantification. WT cells exhibit 3 times larger total cell area compared to *Wdr72* fKO cells. Scale bar, 20 µm. **d** Table quantitative analysis of CD63^+^ vesicle clusters per cell. The number of clusters represents the total CD63^+^ vesicle clusters detected per cell within the ROI. Cluster density was calculated as the number of clusters per micrometer (clusters/µm^2^). Localizations per cluster reflect the number of fluorescence localizations (blinks) assigned to each cluster, and localization density per cluster reports the spatial density of localizations within clusters. Diameter represents the mean cluster diameter

Because WDR72 interacts with the microtubule network, we next examined whether WDR72 influences trafficking along microtubules, especially that associated with multivesicular bodies (MVBs) that carry vesicles destined for extracellular vesicles (EVs). In WT cells, puncta positive for the MVB marker, CD63 (CD63^+^), were widely spread throughout the cytoplasm and often clustered along microtubule (TUBA1A) tracks (Fig. 2a top), aligning with the established role of microtubules in MVB transport [6]. Notably, despite this close spatial relationship, CD63^+^ MVBs showed minimal direct overlap with WDR72 signals on microtubules (Fig. 2a bottom), indicating that WDR72 is not incorporated into MVBs and does not co-traffic with them.

To determine whether WDR72 influences MVB engagement with microtubules, we quantified CD63–TUBA1A colocalized clusters. Loss of WDR72 did not alter the proportion of CD63^+^ vesicles associated with microtubules (Fig. 2b), indicating that MVB–microtubule engagement occurs independently of WDR72.

We next assessed MVB abundance and spatial organization of CD63^+^ vesicles using CODI, a super-resolution nanoimaging clustering analysis program, by quantifying over the full projected cell area (ROI) (Fig. 2c). Quantitative analyses were performed on three independent WT cells and three independent *Wdr72* fKO cells. Cells of each genotype displayed comparable morphology, with no apparent morphological differences between groups (Supplemental Figure S1). For each cell, CD63^+^ cluster number and cluster density (clusters/µm^2^) were calculated, and values are reported as the mean ± SD across cells within each genotype. In CODI, a “localization” is a single detected fluorophore blinking event, and clusters represent groups of localizations arising from fluorophores on one or more proteins within a defined radius (50 nm). Although *Wdr72* fKO cells exhibited a reduced total cell area and consequently fewer total CD63^+^ clusters per cell, normalization to ROI area revealed a higher CD63^+^ vesicle cluster density (clusters/µm^2^) compared to WT cells (Fig. 2d). In contrast, cluster-level properties, including the number of localizations per cluster, localization density within clusters, and cluster diameter, were comparable between WT and *Wdr72* fKO cells, indicating that vesicle size and internal organization are preserved (Fig. 2d).

The preservation of MVB–microtubule association, along with increased intracellular vesicular density, suggests that WDR72 loss of function does not impair MVB engagement with microtubules but rather disrupts steps in MVB transit, such as vesicular transport toward the membrane and export from the cell.

### WDR72 Functional Deficiency Causes Intracellular Retention of Circulating miRNAs

**Fig. 3:**
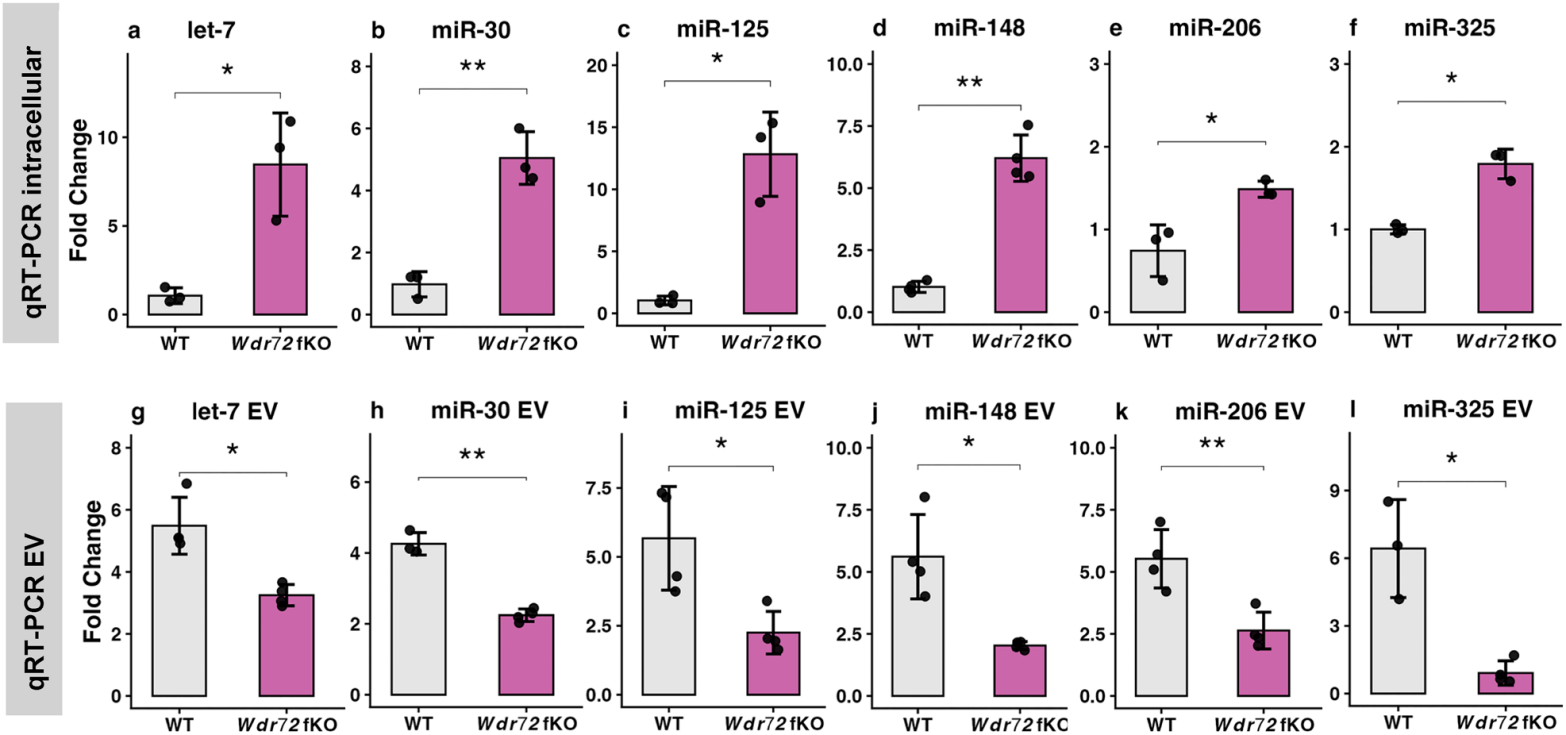
WDR72 loss of function causes intracellular retention and reduced extracellular vesicle export of vesicle-associated miRNAs: **a**–**f** qRT-PCR analysis of vesicular miRNAs in total intracellular RNA isolated from ALCs. Expression levels of let-7 (**a**), miR-30 (**b**), miR-125 (**c**), miR-148 (**d**), miR-206 (**e**), and miR-325 (**f**) are shown as fold change relative to WT. All six miRNAs were significantly elevated in *Wdr72* fKO cells, indicating intracellular accumulation upon loss of WDR72 function. **g**–**l** qRT-PCR analysis of the same miRNAs isolated from extracellular vesicles (EVs) collected from serum-free conditioned media. In contrast to intracellular vesicular miRNAs, the levels of let-7 (**g**), miR-30 (**h**), miR-125 (**i**), miR-148 (**j**), miR-206 (**k**), and miR-325 (**l**) were significantly reduced in the EV from *Wdr72* fKO cells compared to WT, indicating impaired EV-mediated miRNA export. Data are presented as mean ± SD with individual data points representing independent biological replicates. Statistical significance was determined by a two-tailed Welch’s t-test. *P < *0.05*; **P < *0.01*

Because the loss of WDR72 function led to the intracellular accumulation of CD63^+^ vesicles, we next investigated whether vesicle-associated miRNAs were similarly retained within cells. We measured representative circulating miRNAs in both intracellular fractions and extracellular EVs using qRT-PCR. In *Wdr72* fKO cells, intracellular levels of let-7, miR-30, miR-125, miR-148, miR-206, and miR-325 were all significantly increased compared to WT controls (Fig. 3a–f). Conversely, analysis of miRNAs isolated from EVs in conditioned medium showed the opposite pattern: the same six miRNAs were substantially decreased in EVs secreted by *Wdr72* fKO cells (Fig. 3g–l). These opposing changes indicate that WDR72 is essential for the efficient export of circulating miRNAs via EVs and suggest defects in miRNA loading into EVs or release from cell when WDR72 does not function.

### Intracellularly Retained miRNAs Suppress Cytoskeletal and Adhesion-Related Gene Programs

**Fig. 4:**
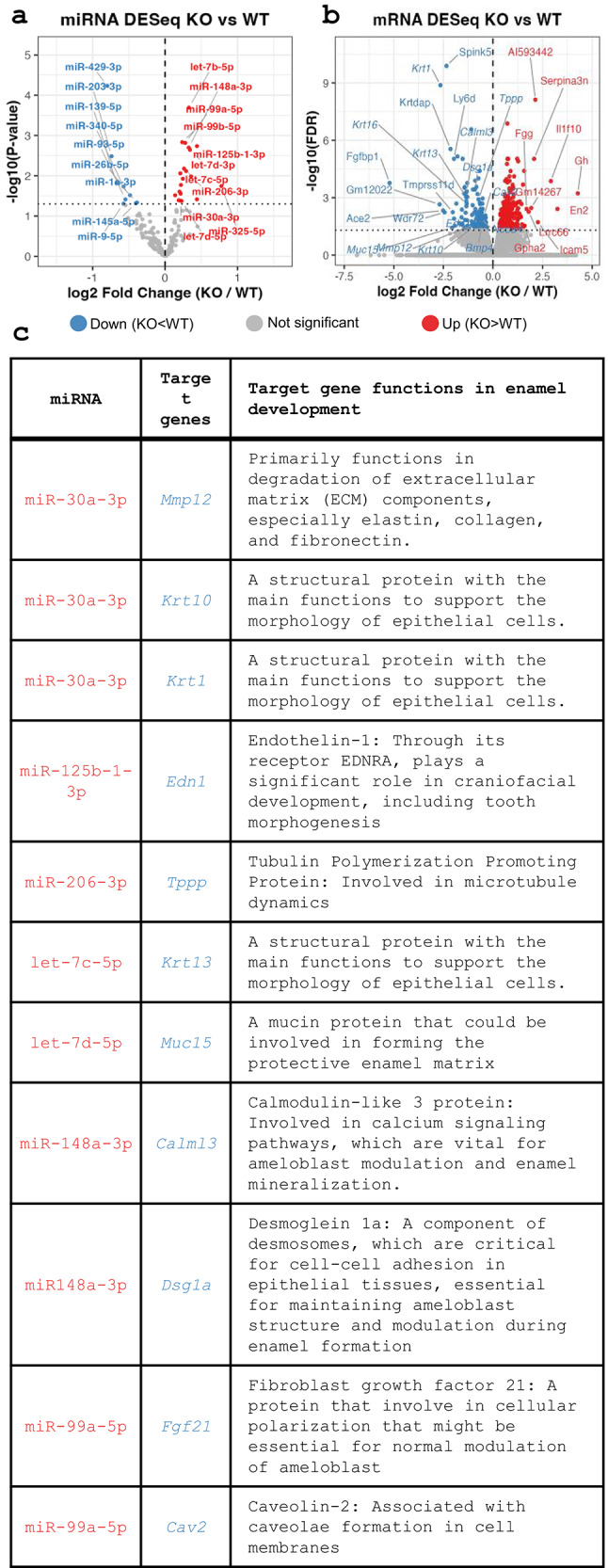
Loss of WDR72 leads to coordinated miRNA upregulation and repression of structural and cytoskeletal mRNA targets in enamel cells: **a** Volcano plot showing differential expression of miRNAs in *Wdr72* fKO versus WT enamel organs. Significantly upregulated miRNAs in *Wdr72* fKO (red) include let-7 family members and miRNAs previously implicated in circulating miRNAs, while downregulated miRNAs are shown in blue. Dashed lines indicate statistical significance and fold-change thresholds. **b** Volcano plot of differentially expressed mRNAs in *Wdr72* fKO versus WT enamel organs. Transcripts significantly downregulated in *Wdr72* fKO (blue), involved in cytoskeletal organization, epithelial structure, vesicle trafficking, and extracellular matrix remodeling, were highlighted, whereas upregulated transcripts are shown in red. **c** Table summarizing integrated regulatory pairs of miRNA–mRNA, significantly altered in miRNA-seq and mRNA-seq, predicted by miRDB. Highlighted targets encode proteins with established roles in enamel development, including cytoskeletal components (*Krt1*, *Krt10*, *Krt13*), extracellular matrix remodeling enzymes (*Mmp12*), calcium signaling regulators (*Calml3*), cell–cell adhesion molecules (*Dsg1a*), microtubule-associated proteins (*Tppp*), and membrane organization factors (*Cav2*). These relationships support an inverse regulatory association between miRNA accumulation and repression of structural gene expression following WDR72 loss of function

Having shown that WDR72 deficiency causes intracellular retention of EV-associated miRNAs in vitro, we then investigated the downstream effects of impaired vesicle-mediate miRNA export on gene expression in vivo. We combined miRNA-seq and previously published mRNA-seq data from maturation-stage enamel organs of *Wdr72* WT and fKO mice [7]. Several miRNAs that accumulated intracellularly in *Wdr72* fKO cells, including miR-30a-3p, miR-125b-1-3p, let-7a-5p, miR-148a-3p, and miR-99-5p, were also significantly increased in the *Wdr72* KO enamel organ, which highlighted in red (Fig. 4a), supporting the biological relevance of the in vitro findings.

Consistent with increased miRNA-mediated repression, volcano plot analysis of mRNA-seq data showed coordinated downregulation of gene groups crucial for ameloblast structure and function that highlighted in blue, including keratins, extracellular matrix remodeling factors, desmosomal components, and cytoskeletal regulators (Fig. 4b). These gene groups are important for maintaining ameloblast polarity, microtubule organization, and cell–cell adhesion, and their suppression is likely to impair microtubule dynamics, weaken desmosomal junctions, and destabilize epithelial morphology—key features of defective ameloblast modulation during enamel maturation.

To test whether intracellular miRNA accumulation is linked to repression of structural gene transcripts, we integrated miRNA and mRNA expression data from *Wdr72* fKO enamel organs with predicted miRNA–mRNA interactions from miRDB. Specifically, we focused on miRNAs significantly upregulated in *Wdr72* fKO enamel organs and examined the expression of their predicted target mRNAs within the same tissue. This analysis revealed a consistent inverse relationship between elevated miRNA levels and reduced expression of their predicted target transcripts. Notably, many of these targets encode structural, cytoskeletal, and adhesion-related proteins (Fig. 4c), supporting the possibility that retention of vesicle-associated miRNAs contributes to repression of gene programs required for normal ameloblast morphology and modulation.

To provide a more complete view of miRNA dysregulation following WDR72 loss of function, we expanded our analysis to include miRNA species that are reduced in *Wdr72* fKO enamel organs (Fig. 4a). Several miRNAs decreased in *Wdr72* fKO samples are predicted to target genes involved in epithelial differentiation, cytoskeletal organization, and extracellular matrix regulation, key processes in ameloblast modulation and enamel maturation. Predicted targets include multiple keratin family members such as: *Krt75*, *Krt35*, *Krt82* as well as gap junction-associated proteins. A comprehensive list of predicted targets for both up- and down-regulated miRNAs is provided in Supplemental Material A.

However, despite the reduced abundance of these miRNAs, their predicted target genes do not show corresponding increases in transcript levels in our mRNA DESeq2 analysis. The full list of significantly altered mRNAs is provided in Supplemental Material B. This suggests that lower abundance of these miRNAs does not directly cause transcriptional changes in cytoskeletal or extracellular matrix genes critical for ameloblast function.

## Discussion

### Direct Association of WDR72 with Microtubules is Required for Microtubule Function During Ameloblast Modulation

The central question driving this study was how WDR72, a protein essential for enamel maturation, is involved in the cellular machinery that supports ameloblast modulation. Ameloblasts are highly dynamic cells that repeatedly change shape as they transition between ruffle- and smooth-ended states, and this modulation depends on efficient intracellular transport. In many cell types, vesicle trafficking occurs along microtubules, which serve as intracellular tracks directing cargo to specific destinations [4–6]. During enamel maturation, this microtubule-dependent transport system is thought to be particularly important, as it enables the rapid vesicle trafficking and cytoskeletal reorganization required for the structural and functional remodeling of ameloblasts. Previous studies have identified WDR72 as a key player in modulating cellular morphology. Our group previously demonstrated that loss of WDR72 disrupts vesicle transport and ruffled end formation in maturation ameloblasts [7], while work by Wang et al. linked WDR72 deficiency to alterations in cytoskeletal organization, particularly involving keratin networks [12]. Together, these observations raised a key unresolved question: through what molecular interactions does WDR72 coordinate vesicle trafficking with cytoskeletal organization during ameloblast modulation?

Given this gap, we questioned whether WDR72 might function in association with the microtubule network. Super-resolution imaging indicated that WDR72 acts more like a microtubule-associated protein than a vesicular component. Although we cannot fully rule out the possibility that the loss of WDR72–microtubule interaction in the *Wdr72* fKO is a secondary effect of microtubule disruption, our data support a specific requirement for functional WDR72 to maintain this association. Importantly, the link between WDR72 deficiency and decreased TPPP levels may explain several of our observations. TPPP is a crucial regulator of microtubule polymerization and was significantly reduced in *Wdr72* fKO ALCs [7] as well as in mRNA-seq analyses of *Wdr72* fKO mouse enamel organs (Fig. 4b). These findings suggest that loss of functional WDR72 is associated with disruption of microtubule regulatory pathways.

Further research is needed to clarify the precise mechanisms of how WDR72 interacts with microtubules; however, our findings suggest that WDR72 plays a role in maintaining a stable microtubule network, likely through direct binding. Supporting WDR72’s involvement in microtubule maintenance, we observed seemingly increased WDR72-positive immunofluorescent signals in *Wdr72* fKO cells compared to WT controls (Fig. 1b). This suggests that the WDR72 protein continues to be produced and may accumulate when its function is impaired. One possible interpretation is that cells attempt to compensate for defective WDR72 activity by increasing protein levels via a feedback mechanism aimed at restoring microtubule functions. However, the persistent diffuse localization and the loss of microtubule engagement show that higher levels of a functionally defective WDR72 are insufficient to restore normal microtubule networks, consistent with a disruption in WDR72-microtubule association. Although our super-resolution analyses were necessarily performed in ameloblast-lineage cells, confirming this interaction in native enamel organ epithelium remains an important goal. Future advances in tissue-compatible super-resolution or other high-resolution imaging methods may allow direct visualization of WDR72-microtubule interactions in vivo.

The peripheral accumulation of WDR72 observed in *Wdr72* fKO cells likely reflects aberrant localization of a nonfunctional protein rather than changes in cell polarity. ALCs do not develop a strong polarization similar to maturation-stage ameloblasts in vivo, and we did not observe consistent co-alignment of WDR72 enrichment with a clear polarity axis. Presumably, the loss of proper interaction with microtubule-associated trafficking components may cause WDR72 to be retained locally or to be recruited in a compensatory way near membrane-associated trafficking zones, resulting in punctate and sometimes regionally concentrated signals despite impaired microtubule-associated organization.

Together, these data position WDR72 within the microtubule transport system and suggest how WDR72 links microtubules to vesicle trafficking during ameloblast modulation. This separation of WDR72 from microtubules provides a consistent explanation for the downstream issues in vesicle export, miRNA retention, and cytoskeletal remodeling seen in *Wdr72* fKO mice.

### Disrupted Vesicular miRNA Export Can Lead to Intracellular miRNA Entrapment and Deregulation of Structural Programs Essential for Enamel Maturation

Vesicle transport along microtubules has been well documented in a variety of cell types [6, 19]. Loss of WDR72 function may impair vesicle movement along microtubule tracks for export. Our nanoimaging data showed that the spatial relationship between vesicles and microtubules was not significantly different between WT and *Wdr72* fKO cells (Fig. 2b). This suggests that vesicular loading onto microtubules remains largely intact in the absence of WDR72 function. Instead, the vesicle export defect appears to occur downstream of initial vesicle–microtubule engagement. Further research is needed to pinpoint the specific step where WDR72 is essential for vesicular export.

What was immediately noticeable in the single-label super-resolution imaging of the MVB marker CD63, followed by cluster-based quantification, was the substantial increase in intracellular vesicle density in *Wdr72* fKO cells. Importantly, parameters related to vesicle size and clustering characteristics remained mostly unchanged, indicating that vesicle biogenesis is not significantly affected by the loss of WDR72 function. Instead, vesicles accumulate inside the cell rather than being effectively transported and exported. Because multivesicular bodies (MVBs) also interact with endo-lysosomal degradation pathways, we cannot rule out that some vesicles may be diverted toward lysosomal processing in WDR72 loss of function [20, 21]. However, our isolation method specifically enriches small extracellular vesicles (< 100 nm), which are mainly involved in miRNA export rather than in heterogeneous degradative vesicles, supporting the idea that impaired vesicle export is the dominant mechanism underlying intracellular vesicles and miRNA retained [20, 21].

If vesicles accumulate intracellularly, their associated cargo would be expected to be retained as well. One major group of vesicle cargo is circulating microRNAs, which are specifically packaged into vesicles and exported to regulate gene expression and maintain intracellular RNA levels. This led us to directly test whether vesicular miRNAs are similarly affected by the loss of WDR72 function. Our qRT-PCR results showed a consistent and opposite pattern of known exporting miRNA distribution between intracellular and exported EVs, depending on the presence of functional WDR72 in ALCs. This inverse pattern strongly indicates that the loss of WDR72 function does not disrupt miRNA production itself. Instead, these findings suggest a defect in vesicular miRNA export, leading to the coordinated retention of miRNAs within the cell and vesicular accumulation. Overall, the increase in miRNAs inside the cell and their decrease outside the cell indicate that functional WDR72 is essential for effective miRNA export–disruption of vesicle transport results in trapping both vesicles and their miRNA cargo within the cell.

Since miRNAs are short (~ 23 nt) antisense RNAs primarily involved in repressing gene expression, the intracellular retention of vesicle-associated miRNAs in *Wdr72* fKO cells likely has direct functional effects. We validated our in vitro findings with in vivo miRNA sequencing from WT and *Wdr72* fKO enamel organs, combined with integrated analysis using miRDB, a curated database for miRNA target prediction and validation.

This integrative analysis is summarized in Fig. 4, which highlights a coordinated inverse relationship between miRNA and mRNA expression in *Wdr72* fKO enamel organs. Multiple circulating miRNAs, including members of the let-7 family, miR-30a, miR-125b, miR-148a, and miR-99a, are significantly upregulated in *Wdr72* fKO enamel organs. In contrast, the corresponding mRNA-seq analysis reveals significant downregulation of transcripts encoding cytoskeletal, adhesion, and vesicle-associated proteins, including keratins (*Krt1*, *Krt10*, *Krt13*), desmosomal components (*Dsg1a*), extracellular matrix regulators (*Mmp12*), and other microtubule-associated factors. Notably, *Tppp* has been previously validated as a direct target of miR-206 [22], and consistent with this relationship, our miRNA sequencing data show that miR-206 is significantly upregulated in both *Wdr72* fKO ALCs and enamel organs (Figs. 3e and 4). This miRNA–mRNA pairing provides a clear mechanistic example of how intracellular retention of circulating miRNAs can directly contribute to the repression of structural regulators when vesicle export is impaired.

Unlike the upregulated miRNAs, which accumulate intracellularly in *Wdr72* fKO cells and are associated with significant repression of their predicted mRNA targets, the reduction of specific miRNA species is unlikely to result directly from vesicle retention. Instead, we interpret these changes as secondary consequences of altered intracellular miRNA homeostasis.

miRNAs share common biogenesis and effector machinery, including Drosha/Dicer processing pathways and Argonaute-containing RNA-induced silencing complexes (RISC). Expansion of specific miRNA populations can therefore indirectly reduce the effective abundance or activity of other miRNAs through competition for shared processing factors, Argonaute loading, or interactions within competing endogenous RNA networks [23–25]. Such competitive effects are generally tolerated due to extensive functional redundancy within miRNA regulatory networks, in which individual miRNAs regulate multiple targets, and individual genes are often controlled by multiple miRNAs [26, 27].

Consistent with this framework, reduced miRNA species in *Wdr72* fKO enamel organs do not exhibit a direct inverse relationship with mRNA abundance of their predicted targets, suggesting that their downregulation reflects homeostatic redistribution of miRNA machinery rather than primary regulatory events. In contrast, upregulated miRNAs, whose intracellular accumulation is consistent with impaired vesicle-mediated export, show strong correspondence with repression of their predicted targets, supporting a direct regulatory role.

Together, these data indicate that loss of WDR72 disrupts vesicle-mediated miRNA export, leading to increased intracellular miRNA retention that perturbs both primary regulatory pathways (direct repression of structural target transcripts) and secondary compensatory networks (redistribution of miRNA abundance through shared biogenesis and effector machinery). This altered miRNA landscape appears to shift structural gene expression programs, particularly those governing ameloblast polarity, cytoskeletal integrity, and the cyclical shape changes required for ameloblast modulation, thereby contributing to defective ameloblast function and impaired enamel maturation in *Wdr72* fKO mice.

## Conclusion

Overall, our findings suggest a model in which WDR72 acts as a microtubule-associated regulator that supports vesicular movement and export, as well as miRNA crosstalk. When the WDR72 function is disrupted, vesicles accumulate intracellularly, miRNA export is blocked, and the accumulated miRNAs suppress gene networks essential for cytoskeletal changes. This sequence of events accounts for the vesicular trafficking issues, cytoskeletal problems, and regulatory failure seen in WDR72 functionally deficient ameloblasts. By connecting WDR72 to microtubule-dependent vesicle transport and miRNA regulation, this study introduces a new layer of post-transcriptional control in ameloblast function. More broadly, these results demonstrate how impairing vesicular-based miRNA transport can weaken epithelial structure and function, offering new insights into the cellular processes involved in enamel maturation.

## Supplementary Information

Below is the link to the electronic supplementary material.

Supplementary file1 (PDF 268 kb)

Supplementary file2 (XLSX 30 kb)

Supplementary file3 (XLSX 151 kb)

Supplementary file4 (XLSX 582 kb)

## Data Availability

All data of this study are available from the corresponding author upon reasonable request.
