# Supplementary material for: WDR72 Regulates Microtubule-Associated Vesicular miRNA Export in Ameloblasts During Enamel Maturation
Source: Calcif Tissue Int. 2026 Apr 27;117(1):68. doi: 10.1007/s00223-026-01521-x (PMC13121283; doi:10.1007/s00223-026-01521-x)
Supplement: Supplementary file 1 — Supplementary file1 (PDF 268 kb) [file 223_2026_1521_MOESM1_ESM.pdf]

# Figure S1

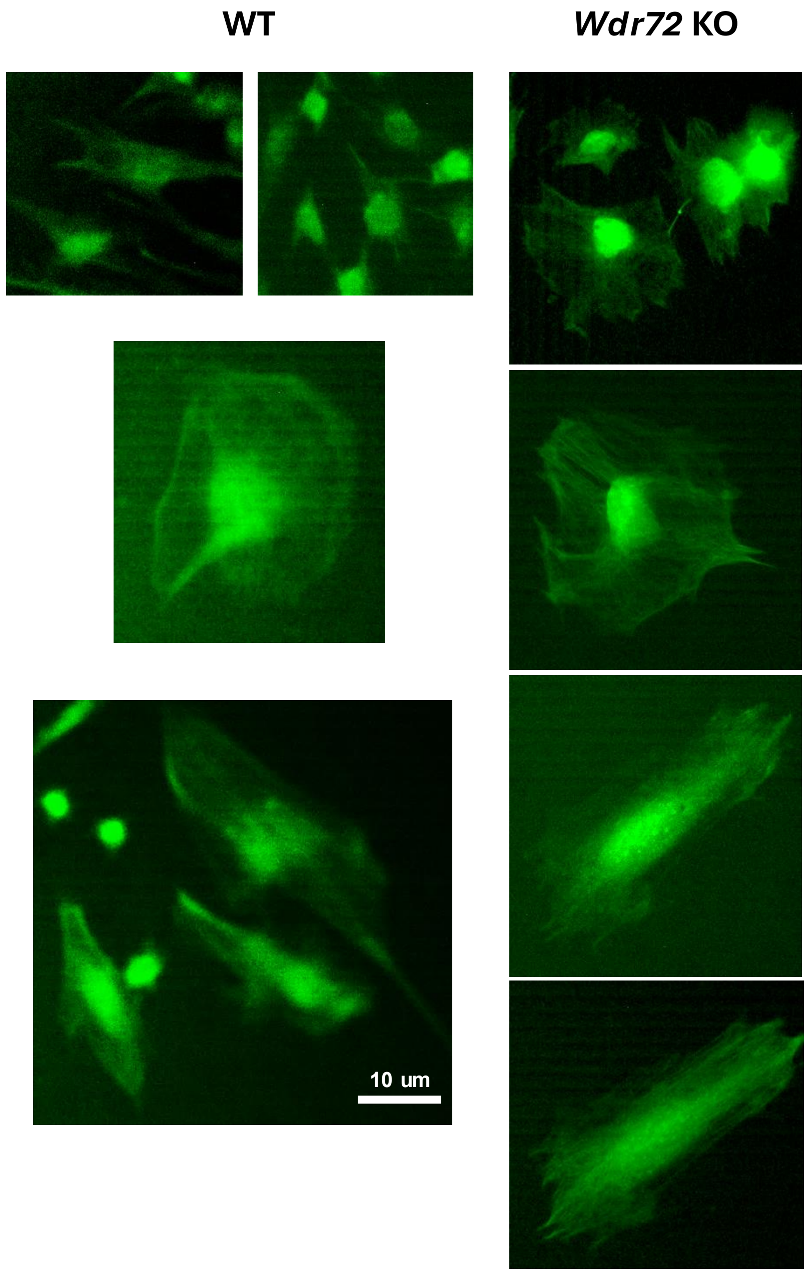


**Supplemental Figure S1. Low-resolution live-cell imaging of microtubule organization in WT and *Wdr72* fKO ameloblast-lineage cells (ALCs).** WT and *Wdr72* fKO ALCs were stained with Tubulin Tracker™ Green (Thermo Fisher Scientific, Cat. No. T34078) to visualize microtubules under live-cell conditions. A 1x working staining solution was prepared in PBS supplemented with probenecid according to the manufacturer's instructions, and cells were washed twice with PBS prior to incubation. Cells were incubated with the 1x Tubulin Tracker solution (600 µL per well in 2-well chamber slides or 200 µL per well in 8-well chamber slides) at 37°C for 30 minutes. Live-cell imaging was performed immediately after incubation using an Olympus CKX53 inverted microscope equipped with Infinity 3S software and a 40x objective. Representative fields are shown.
